# Association between low-grade albuminuria and frailty among community-dwelling middle-aged and older people: a cross-sectional analysis from I-Lan Longitudinal Aging Study

**DOI:** 10.1038/srep39434

**Published:** 2016-12-21

**Authors:** Chun-Chin Chang, Chien-Yi Hsu, Ting-Yung Chang, Po-Hsun Huang, Li-Kuo Liu, Liang-Kung Chen, Jaw-Wen Chen, Shing-Jong Lin

**Affiliations:** 1 Taipei Veterans General Hospital Taoyuan Branch, Taoyuan, Taiwan; 2 Cardiovascular Research Center, National Yang-Ming University, Taipei, Taiwan; 3 Institute of Clinical Medicine, National Yang-Ming University, Taipei, Taiwan; 4 Department of Internal Medicine, School of Medicine, College of Medicine, Taipei Medical University, Taipei, Taiwan; 5 Division of Cardiology and Cardiovascular Research Center, Department of Internal Medicine, Taipei Medical University Hospital, Taipei, Taiwan; 6 Division of Cardiology, Department of Medicine; Taipei Veterans General Hospital, Taipei, Taiwan; 7 Center for Geriatrics and Gerontology, Taipei Veterans General Hospital, Taipei, Taiwan; 8 Aging and Health Research Center, National Yang-Ming University, Taipei, Taiwan; 9 Institute of Public Health, National Yang-Ming University, Taipei, Taiwan; 10 Department of Medical Research; Taipei Veterans General Hospital, Taipei, Taiwan; 11 Institute of Pharmacology, National Yang-Ming University, Taipei, Taiwan

## Abstract

Frailty is characterized by decreased physiological reserve and increased vulnerability to atherosclerosis and subsequent mortality. Recently, low-grade albuminuria has been proposed as an atherosclerotic risk factor. We aimed to investigate the relationship between low-grade albuminuria and frailty by using cross-sectional data among community-dwelling middle-aged and older people. Totally, 1,441 inhabitants of I-Lan County with normal urinary albumin excretion (urine albumin to urine creatinine ratio [UACR] <30 mg/g) were enrolled (677 men; mean age 63 ± 9 years, range from 50 to 91 years old). Assessment of frailty was based on the ‘Fried frailty phenotype’ criteria, including weight loss, grip strength, exhaustion, slowness and low physical activity. The study population was stratified into quartiles according to UACR levels. Age, body mass index, hypertension, diabetes, systolic blood pressure, insulin resistance, fasting glucose and high-sensitivity C-reactive protein levels were increased with the increment of UACR (P for trend <0.05). The prevalence of prefrailty/frailty and its components increased across the UACR quartiles. A multivariate stepwise logistic regression analysis revealed that UACR was independently associated with the likelihood of prefrailty/frailty (odds ratio 1.13, 95% CI 1.01–1.27). In conclusion, low-grade albuminuria is associated with the increased prevalence of prefrailty/frailty.

Frailty, a highly prevalent medical syndrome in elder population, is characterized as decreased physiological reserve and enhanced vulnerability for morbidity and mortality^1^,^2^. The core clinical presentations of frailty include unintentional weight loss, loss of muscle mass, weakness, poor endurance and decreased physical activity^3^. While developing progressively, frailty may render the elderly further vulnerable with increased cardiovascular burden. Data from observational studies have indicated a significant correlation between frailty and risk of cardiovascular disease and mortality in elderly men and women^4^,^5^.

The presence of microalbuminuria, defined as a urine albumin-to-creatinine ratio (UACR) of 30 to 300 mg/g, is associated with progression of atherosclerotic vascular disease, elevated levels of inflammation markers, increased risk of osteoporotic fracture and the risk of cardiovascular events in both diabetic and nondiabetic individuals^6^,^7^,^8^,^9^. Increasing evidence has shown that low-grade albuminuria, defined as UACR as 0 to 30 mg/g, an earlier stage with UACR below the microalbuminuria threshold, is also associated with an increased risk of incident cardiovascular disease and all-cause mortality^10^,^11^,^12^. The Heart Outcomes Prevention Evaluation (HOPE) study found a continuous association between levels of UACR and cardiovascular events^6^. Major cardiovascular events increased by 5.9% for every 3.0 mg/g increase in UACR, starting at the UACR level of 4.4 mg/g. Moreover, population studies showed that the incidence of albuminuria increase with advancing age, even in the absence of diabetes, hypertension, or chronic kidney disease^13^. However, no prior studies to date have looked for a relationship between albuminuria and frailty.

The aim of the present study is to investigate the relationship between low-grade albuminuria and frailty. We tested the hypothesis that the degree of low-grade albuminuria is associated with the status of frailty by using cross-sectional analysis among a community-based cohort (ILAS cohort) in Taiwan.

## Results

Total 1,839 inhabitants of I-Lan County of Taiwan were enrolled. After further excluding participants who had a UACR level exceeding normal range (30 mg/g creatinine, n = 398), 1,441 participants remained eligible. A total of 1,441 participants of the I-Lan Longitudinal Aging Study (ILAS) were analyzed (677 men, 47%; mean age 63 ± 9 years, range from 50 to 91 years old). In the study participants, 37% study subjects had hypertension, 13% had diabetes, and 5% had coronary artery disease. Assessment of frailty was based on the ‘Fried frailty phenotype’ criteria, including weight loss, grip strength, exhaustion, slowness and low physical activity. All study participants were divided into two groups according to the frailty status: 804 (56%) were non-frail, 637 (44%) were prefrail or frail.

Table 1 summarizes the demographic and clinical characteristics of the study subjects. In participants with prefrail or frail status, they were older, had higher waist circumference, had more histories of hypertension, diabetes, and higher systolic blood pressure (SBP), serum levels of homocystein, high-sensitivity C-reactive protein (hs-CRP) and UACR, but significantly lower estimated glomerular filtration (eGFR), high-density lipoprotein cholesterol (HDL-C), low-density lipoprotein cholesterol (LDL-C) and bone mineral density (BMD) than those were non-frail (all p < 0.05).

**Figure 1:**
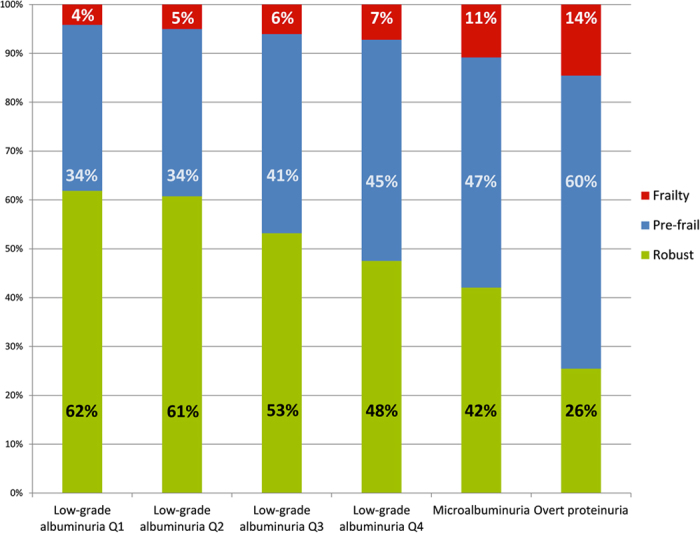
The prevalence of frailty at different stages of proteinuria in ILAS cohort.

The prevalence of frailty at different stages of proteinuria of the ILAS cohort (total 1,839 participants) was shown in Fig. 1. To further clarify the relationship between low-grade albuminuria and frailty, the study population was then stratified into quartiles according to UACR levels. The clinical and biochemical parameters according to the quartile groups for UACR were demonstrated in Table 2. There were no significant differences among the four groups for renal function (eGFR), carotid intima media thickness, lipid profiles (including TC, triglycerides [TG], LDL-C, HDL-C), and serum level of homocystein. However, traditional risk factors including age, body mass index (BMI), hypertension, diabetes, SBP, homeostasis model assessment-estimated insulin resistance (HOMA-IR), fasting glucose concentration and hs-CRP level were significantly increased with the increment of UACR. In contrast, BMD was decreased with the increment of UACR (all P for trend <0.05).

**Figure 2:**
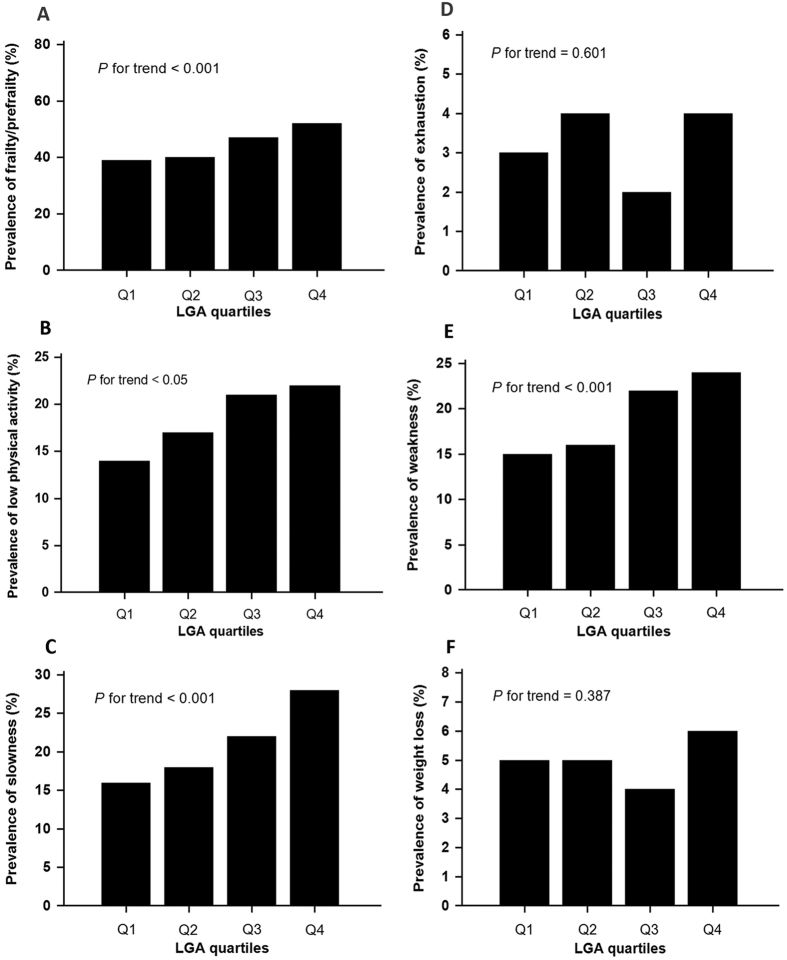
Prevalence of prefrailty/frailty (Panel A), low physical activity (Panel B), slowness (Panel C), exhaustion (Panel D), weakness (Panel E), weight loss (Panel F) in different UACR quartiles.

The prevalence of prefrailty/frailty and its components in the different UACR quartiles were presented in Fig. 2 and Table 3. From the lowest UACR quartile across to the highest one, the prevalence of prefrailty/frailty was 39, 40, 47, and 52%, respectively (*P* for trend <0.001). Similarly, the prevalence of frailty components including low physical activity, weakness and slowness increased significantly with the increment of UACR. There was no significant difference for exhaustion and body weight loss among the four groups.

**Figure 3:**
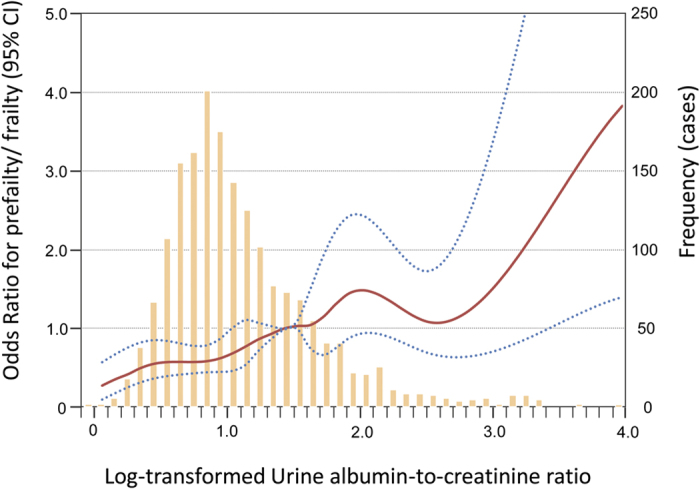
Odds ratios for incident frailty according to log-transformed urine albumin-to-creatinine ratio (Log UACR).

As shown in Table 4, univariate logistic regression analysis revealed that age, current smoking, hypertension, diabetes, LDL-C, HDL-C, homocystein, BMD and UACR were significantly associated with prefrailty/frailty. After performing multivariable stepwise logistic regression analysis, age and UACR were positively associated with prefrailty/frailty, while HDL-C and BMD were inversely associated with prefrailty/frailty. The odds ratios (ORs) for incident frailty according to log-transformed urine albumin-to-creatinine ratio (Log UACR) in the ILAS cohort was shown in Fig. 3.

**Figure 4:**
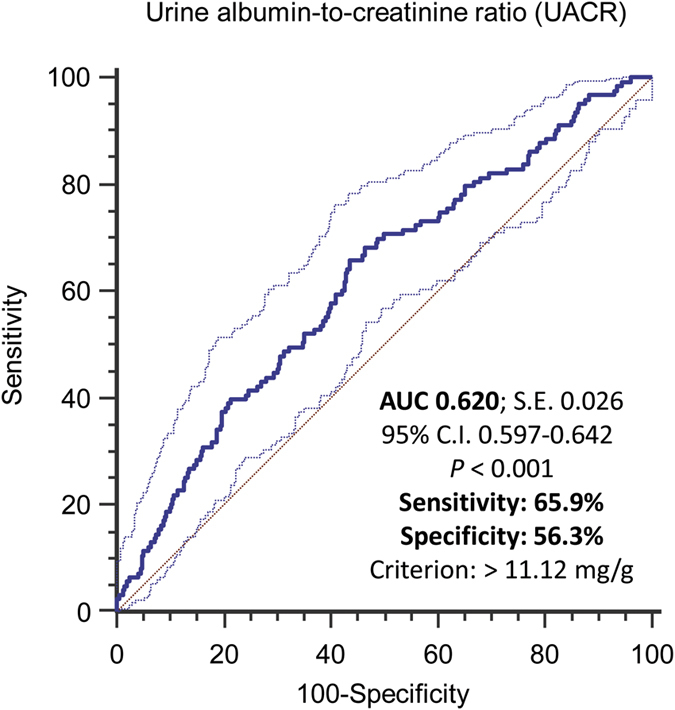
The receiver operating characteristic (ROC) curve analysis for the diagnosis of frailty by using urine albumin-to-creatinine ratio (UACR) as predictor.

The prevalence of prefrailty/frailty was gradually elevated according to the UACR quartiles (odds ration were 1.13, 1.45 and 2.15 for UACR quartile 2, 3 and 4 compared with the lowest quartile, *P* for trend <0.001). As shown in Fig. 4, the receiver operating characteristic (ROC) analysis was performed to assess the predictive accuracy of UACR in the diagnosis of frailty. The optimal cutoff point of UACR was determined according to maximal Youden index. The optimal cutoff points determined for UACR were 11.12 mg/g (sensitivity 65.9%, specificity 53.3%). Further subgroup analysis showed the prevalence of prefrailty/frailty was also increased significantly along with the increments of UACR quartiles in most dichotomous subgroups (Table 5). UACR displayed a stronger association with prefrailty/frailty in older (≥65 year), male, obese (BMI ≥ 24 kg/m^2^), non-diabetic and non-hypertensive individuals.

## Discussion

The main question addressed by the present study was whether UACR below the current microalbuminuria threshold was associated with prefrailty/frailty in elderly adults. Our study first demonstrated that low-grade albuminuria was significantly associated with prefrailty/frailty after adjusting other risk factors. Clinical significance of this relationship may result in earlier surveillance of low-grade albuminuria, and identify high-risk population to be frail.

Microalbuminuria is a well-known cardiovascular risk indicator in both diabetic and nondiabetic individuals^14^,^15^,^16^. Microalbuminuria is associated with low-grade systemic inflammation and reflects vascular damage in the glomerulus and systemic endothelial dysfunction. A large follow-up study over 4.4 years enrolled 2089 non-diabetic subjects, which demonstrated a positive association between all-cause mortality and urine albumin excretion^17^. The lowest UACR level associated with increased relative risk (RR) for mortality was the 60th percentile (≥6.7 mg/g, RR 2.4; 95% CI: 1.1–5.2). In the HOPE cohort following up individuals without diabetes, there was a continuous association between albuminuria and cardiovascular events extending at least as low as 4.4 mg/g^6^. Undoubtedly, the normal range of UACR has increasingly being challenged during the past decade. Low-grade albuminuria, an indicator of glomerular endothelial dysfunction, is believed as an important marker of future cardiovascular events^18^,^19^. The PREVEND study showed a positive dose-response relationship between increments of urinary albumin excretion and mortality^16^. The relationship was already apparent at levels of albuminuria below the current threshold. Consistent result is also reported in apparently healthy population. Data from the Framingham Heart Study, a community-based sample of nonhypertensive and nondiabetic individuals, demonstrated that the low grade albuminuria is an independent predictor for an increased incidence of cardiovascular events in healthy subjects^10^.

Frailty, a prevalent geriatric syndrome, is believed to be a complex process involving multiple systems, particularly in cardiovascular system^20^,^21^. The Healthy Aging and Body Composition Study showed that frailty was also a risk factor for the development of incident cardiovascular disease^22^. Investigators have proposed that frailty may lead to atherosclerotic disease, and atherosclerotic disease may subsequently lead to frailty. Furthermore, pre-frailty, which is potentially reversible, has been showed is independently associated with a higher risk of older adults developing cardiovascular disease^5^. Among the five Fried criteria of frailty, slowness (low gait speed) seems to be the best single predictor of future cardiovascular disease. Slowness could reflect subclinical cardiovascular disease, such as thickened carotid intima-media, carotid plaque, left ventricular hypertrophy and abnormal ankle brachial index^23^,^24^. Our data, consistent with these findings, indicate slowness was the most significant parameter which was associated with the increment of UACR among the five criteria.

The pathophysiological link between low-grade albuminuria and frailty might be attributed to the shared cardiovascular risk factors. In our study, the prevalence of hypertension and SBP increased with the increment of UACR indeed. However, after further adjustment, the common cardiovascular risk factors such as blood pressure, blood sugar, and blood cholesterol, and the inflammatory biomarkers such as hs-CRP and homocystein both failed to link to the presence of frailty, but the association of low-grade albuminuria and frailty persisted, suggesting that additional mechanisms may be involved beyond the effects of cardiovascular disease. Besides, in subgroup analysis, the positive association between low-grade albuminuria and frailty was more significant in nondiabetic and nonhypertensive individuals. Taken together, it seems that low-grade albuminuria might be directly linked to the presence of frailty via a novel mechanism. On the other hand, though less likely, we cannot exclude the possibility that low-grade albuminuria may be a result of frailty. Future prospective studies are warranted to elucidate the causal relationship between low-grade albuminuria and frailty.

Interestingly, our study demonstrated that lower BMD was associated with prefrailty/frailty status. In a recent study, Barzilay *et al*. have reported an association between albuminuria and hip fracture^9^. The authors suggested that albuminuria, a renal microvascular disorder, contributes to hip fracture risk in older adults. Hence, a comprehensive survey of albuminuria and maintaining bone health are important while prefrailty or frailty is identified.

Some possible limitations should be mentioned in this study. First, we cannot establish the causal relationship by the inherent limitation of this cross-sectional design of the study. Second, our participants were relatively healthier than community dwelling older adults because we excluded participants with any disability. Therefore, our study results might underestimate the prevalence of frailty and its associated health impact in the community. However, the current findings in a less-illed and ambulant cohort did provide a chance to elucidate the potential role of low-grade albuminuria for the presence of frailty. Furthermore, the existence of low-grade albuminuria may imply the presence of frailty while other common risk factors such as hypertension and diabetes were attenuated or absent. Though the current findings suggest the significance of low-grade albuminuria for hidden frailty, future prospective study will be needed to confirm whether low-grade albuminuria rather than frailty could be an independent predictor of long-term cardiovascular outcomes. Then, it could be possible to define if low-grade albuminuria should be addressed in therapeutic strategies and which cut-off threshold should be used in clinical practice.

In conclusion, our study supports the hypothesis that very low degree of urinary albumin excretion below the threshold for microalbuminuria is associated with frailty in a relatively healthy middle-aged and elderly cohort. Low-grade albuminuria rather than the classic risk factors could be a novel marker of frailty, suggesting the unique mechanisms for the development of frailty in elderly people. Hopefully, it may enable clinicians to provide timely interventions with aggressive lifestyle modification and adjust medical therapy to withhold the progression of frailty in individuals with or without significant cardiovascular diseases.

## Methods

### Study design and population

ILAS is a community-based aging cohort study in I-Lan County of Taiwan^24^. Community-dwelling adults over 50 years old were randomly sampled through the household registrations of the county government in Yuanshan Township of I-Lan County. Selected residents were invited to participate from the research team, and were enrolled when they had fully consented and agreed for participation. The inclusion criteria were: (i) inhabitants of I-Lan County without a plan to move in the near future; and (ii) inhabitants over 50 years old. Any respondents that met any one of the following conditions were excluded from the study: (i) unable to communicate with the interviewer; (ii) poor function status which could lead to a fail in evaluation; (iii) limited life expectancy (<6 months) because of major illnesses; (iv) currently institutionalized people. This research was conducted according to the principles expressed in the Declaration of Helsinki. All participants had given their written informed consents, and the study was approved by the Institutional Review Board of the National Yang-Ming University, Taipei, Taiwan.

### Demographic and physical examinations

Medical history, including information about conventional cardiovascular risk factors (smoking, hypertension, diabetes mellitus, hyperlipidemia, peripheral artery disease, coronary artery disease, and chronic kidney disease), previous cardiovascular events (myocardial infarction and cerebrovascular disease), and current drug treatment was obtained during a personal interview and from medical files. Weight, height, and waist circumference were measured and BMI was calculated. Brachial blood pressure was accessed with a mercury sphygmomanometer after patients sat for 15 minutes or longer. The average of three SBP measurements was used for the analysis.

### Definition of frailty

In the present study, frailty was defined according to Cardiovascular Health Study frailty index that Fried proposed^3^. Cardiovascular Health Study frailty index was constructed from five criteria that included weight loss, exhaustion, slowness, weakness and low physical activity. Weight loss was defined as self-reported unintentional loss of 10 pounds in prior year. Exhaustion was identified according to two questions from the Center for Epidemiologic Studies Depression Scale. Slowness was determined according to quintile of the 6-m gait speed test adjusted for sex and height. Participants answering “yes” to the question: “Do you have difficulty rising from a chair?” were categorized as frail for weakness. Low physical activity was defined as not performing daily leisure activities such as walking or gardening or exercising at less than once a week. Frail was classified as the presence of three of the five criteria. One or two criteria were classified as prefrailty and none was defined as non-frailty.

### Urinary albumin excretion

A single voided morning urine sample was used to measure the UACR (mg/g). UACR measured in a spot urine sample is highly correlated with 24-hour urine albumin excretion^25^,^26^,^27^. The specific cut-off points of UACR quartiles were as follows: quartile 1 (Q1): 0–4.82 mg/g; quartile 2 (Q2):4.83–7.67 mg/g; quartile 3 (Q3):7.68–12.7 mg/g; quartile 4 (Q4):12.8–29.95 mg/g.

### Laboratory measurement

All blood samples were drawn with the participant in the seated position after a 10-h overnight fast. Serum concentrations of glucose, TC, TG, LDL-C and HDL-C were determined using an automatic analyzer (ADVIA 1800, Siemens, Malvern, PA, USA). Whole-blood glycated hemoglobin A1c (HbA1c) was measured by an enzymatic method using the Tosoh G8 HPLC Analyzer (Tosoh Bioscience, Inc., San Francisco, CA, USA). The serum levels of hs-CRP, homocystein and insulin-like growth factor-1 were also measured.

### Statistical analysis

The analysis was performed on the complete data set, and results were expressed as mean ± SD or as percent frequency. Comparisons between two groups were made by paired or unpaired Student t test, Mann-Whitney U test, or Chi square test, as appropriate. Comparisons of continuous variables among three groups were performed by analysis of variance (ANOVA). Subgroup comparisons of categorical variables were assessed by Chi square test. Logistic regression analysis was performed to evaluate the association between frailty status and several potential risk factors. We further used Log-transformed UACR linear splines (knots at intervals of 0.1 Log UACR between 0 and 4) in logistic regression models, providing ORs with UACR of 30 mg/g (by definition the lower limit of microalbuminuria) selected as a reference. Reference ranges are important for statistical testing, but they do not alter the shape of the association across the full range of exposure. The ROC analysis was performed to assess the predictive accuracy of UACR in the diagnosis of frailty. Data were analyzed using SPSS software (version 20, SPSS, Chicago, Illinois, USA). A p-value of less than 0.05 was considered to indicate statistical significance.

## Additional Information

**How to cite this article**: Chang, C.-C. *et al*. Association between low-grade albuminuria and frailty among community-dwelling middle-aged and older people: a cross-sectional analysis from I-Lan Longitudinal Aging Study. *Sci. Rep.* **6**, 39434; doi: 10.1038/srep39434 (2016).

**Publisher's note:** Springer Nature remains neutral with regard to jurisdictional claims in published maps and institutional affiliations.

## Figures and Tables

**Table 1:** Comparison of general characteristics between different frail status.

	Non-frail (n = 804)	Prefrail or Frail (n = 637)	*p* value
Age, years	60.6 ± 7.3	66.8 ± 9.5	<0.001
Sex, men	364 (45)	313 (49)	0.151
BMI	24.6 ± 3.4	24.6 ± 3.4	0.906
Waist circumference, cm	83.4 ± 9.2	85.0 ± 9.6	0.001
Current smoking	125 (16)	124 (20)	0.058
Hypertension	258 (32)	268 (42)	<0.001
Diabetes	88 (11)	100 (16)	0.009
CAD	31 (4)	34 (5)	0.202
SBP, mmHg	127.4 ± 15.7	130.1 ± 16.7	0.001
Fasting glucose, mg/dl	98.4 ± 18.3	100.8 ± 25.8	0.058
eGFR, ml/min	86.8 ± 26.2	77.1 ± 28.4	<0.001
HDL-C, mg/dl	56.2 ± 13.9	53.9 ± 13.6	0.002
LDL-C, mg/dl	121.3 ± 32.5	117.7 ± 32.7	0.038
Triglyceride, mg/dl	117.2 ± 80.9	122.5 ± 72.6	0.193
Uric acid, mg/dl	5.8 ± 1.5	5.9 ± 1.5	0.061
Homocystein, μmol/l	12.4 ± 4.68	13.7 ± 6.23	<0.001
hs-CRP, mg/dl	0.19 ± 0.35	0.24 ± 0.5	0.044
IGF-1, ng/ml	144.9 ± 58.6	127.2 ± 52.8	<0.001
HOMA-IR, unit	1.96 ± 1.61	2.01 ± 1.92	0.654
BMD, g/cm^2^	0.86 ± 0.13	0.81 ± 0.14	<0.001
UACR, mg/g creatinine	8.98 ± 6.12	10.53 ± 6.95	<0.001

Values are mean ± SD or n (%), BMI: body mass index, BMD: bone mineral density, CAD: coronary artery disease, eGFR: estimated glomerular filtration rate, hs-CRP: high-sensitivity CRP, HDL: High-density lipoprotein, HOMA-IR: homeostasis model assessment-estimated insulin resistance, IGF-1: insulin like growth factor-1, LDL: Low-density lipoprotein, SBP: systolic blood pressure, UACR: urine albumin-to-creatinine ratio.

**Table 2:** Comparison of general characteristics between different stages of UACR.

	LGA Q1 (n = 356)	LGA Q2 (n = 354)	LGA Q3 (n = 368)	LGA Q4 (n = 363)	*P* value	*P* for trend
UACR, mg/g	3.4 ± 0.9	6.1 ± 0.8	9.7 ± 1.5	19.2 ± 5	<0.001**	<0.001**
Age, year	61.3 ± 8.5	62.5 ± 8.6	64 ± 9.0	65.5 ± 9.2	<0.001**	<0.001**
Male	227 (64)	162 (46)	134 (36)	154 (42)	<0.001**	<0.001**
BMI	24.4 ± 3.1	24.3 ± 3	24.7 ± 3.6	25.1 ± 3.8	0.007*	0.002*
Hypertension	105 (30)	113 (32)	146 (40)	162 (45)	<0.001**	<0.001**
Diabetes	33 (9.3)	42 (11.9)	52 (14.1)	61 (16.8)	0.020*	0.002*
SBP, mmHg	124 ± 15	126 ± 15	130 ± 16	134 ± 17	<0.001**	<0.001**
Triglycerides, mg/dl	118 ± 91	114 ± 66	121 ± 72	126 ± 79	0.208	0.089
Cholesterol, mg/dl	194 ± 32	195 ± 35	199 ± 36	196 ± 35	0.169	0.187
HDL-C, mg/dl	54 ± 14	55 ± 14	56 ± 14	55 ± 14	0.243	0.532
LDL-C, mg/dl	119 ± 30	119 ± 33	121 ± 35	119 ± 33	0.810	0.655
Fasting glucose, mg/dl	97 ± 15	98 ± 17	100 ± 21	103 ± 30	0.001**	<0.001**
HOMA-IR	2.0 ± 1.8	2.0 ± 1.4	2.2 ± 1.9	2.5 ± 2.4	0.001**	<0.001**
eGFR, ml/min	82.1 ± 23	84 ± 26	84 ± 29	82 ± 31	0.009*	0.111
cIMT, mm	0.68 ± 0.1	0.68 ± 0.1	0.69 ± 0.1	0.70 ± 0.1	0.229	0.064
hs-CRP, mg/dl	0.18 ± 0.4	0.19 ± 0.3	0.26 ± 0.6	0.23 ± 0.4	0.081	0.043*
Homocystein, μmol/l	12.9 ± 5.6	12.4 ± 3.7	13.2 ± 6.6	13.4 ± 5.4	0.069	0.086
BMD, g/cm^2^	0.87 ± 0.13	0.84 ± 0.13	0.83 ± 0.13	0.82 ± 0.14	<0.001**	0.004

Values are mean ± SD or n (%), ^*^P < 0.05, ^**^P < 0.001, BMD: bone mineral density, BMI = body mass index, cIMT: carotid intima media thickness, eGFR: estimated glomerular filtration rate, hsCRP: high-sensitivity CRP, HDL: High-density lipoprotein, HOMA-IR: homeostasis model assessment-estimated insulin resistance, LGA: low-grade albuminuria, LDL: Low-density lipoprotein, UACR: urine albumin-to-creatinine ratio, Q1: quartile 1, Q2: quartile 2, Q3: quartile 3, Q4: quartile 4.

**Table 3:** Comparison of frail status between different stages of UACR.

	LGA Q1 n = 356	LGA Q2 n = 354	LGA Q3 n = 368	LGA Q4 n = 363	*P* value	*P* for trend
Prefrail/frail	137 (39)	140 (40)	171 (47)	189 (52)	0.001*	<0.001**
Exhaustion	9 (3)	15 (4)	9 (2)	14 (4)	0.414	0.601
Low physical activity	61 (17)	50 (14)	78 (21)	79 (22)	0.026*	0.022*
Weakness	55 (15)	58 (16)	81 (22)	86 (24)	0.009*	0.001*
Slowness	58 (16)	62 (18)	81 (22)	103 (28)	<0.001**	<0.001**
Weight loss	16 (5)	19 (5)	16 (4)	23 (6)	0.597	0.387
CHS frailty score	0.56 ± 0.83	0.58 ± 0.86	0.72 ± 0.93	0.84 ± 0.99	<0.001**	<0.001**

Values are mean ± SD or n (%), ^*^P < 0.05, ^**^P < 0.001, CHS: Cardiovascular Health Study, LGA: low grade albuminuria, Q1: quartile 1, Q2: quartile 2, Q3: quartile 3, Q4: quartile 4.

**Table 4:** Binary logistic regression analysis of risk factors associated with frailty and prefrailty.

	Univariate		Multivariate	
	Odds Ratio (95% CI)	P value	Odds Ratio (95% CI)	P value
Age, 1 SD = 9.0 year	2.14 (1.90–2.40)	<0.001**	1.98 (1.75–2.24)	<0.001**
Smoking (yes or no)	1.42 (1.13–1.78)	0.003*	—	—
Hypertension (yes or no)	1.54 (1.24–1.91)	<0.001**	—	—
Diabetes (yes or no)	1.52 (1.11–2.06)	0.008*	—	—
LDL-C, 1 SD = 33 mg/dl	0.89 (0.80–0.99)	0.039*	—	—
HDL-C, 1 SD = 14 mg/dl	0.84 (0.76–0.94)	0.002*	0.87 (0.77–0.98)	0.023*
Homocystein, 1 SD = 5.5	1.36 (1.19–1.56)	<0.001**	—	—
HOMA-IR, 1 SD = 1.9 unit	1.02 (0.92–1.13)	0.646	—	—
UACR, 1 SD = 6.5 mg/g creatinine	1.27 (1.14–1.41)	<0.001**	1.13 (1.01–1.27)	0.033*
BMD, 1 SD = 0.17 g/cm^2^	0.82 (0.74–0.91)	<0.001**	0.88 (0.78–0.98)	0.025*

^*^P < 0.05, ^**^P < 0.001, BMD: bone mineral density, HDL: High-density lipoprotein, HOMA-IR: homeostasis model assessment-estimated insulin resistance, LDL: Low-density lipoprotein, UACR: urine albumin-to-creatinine ratio.

**Table 5:** Elevated LGA groups associated with the prevalence of prefrailty/frailty in total and stratified population.

		UACR groups OR (95% CI)				
		LGA Q1	LGA Q2	LGA Q3	LGA Q4	P for trend
Total		1.00	1.13 (0.87–1.47)	1.45 (1.11–1.89)	2.15 (1.36–3.40)	<0.001**
Age	<65 yr	1.00	1.03 (0.73–1.45)	1.04 (0.73–1.49)	1.14 (0.57–2.26)	0.710
	≥65 yr	1.00	1.09 (0.69–1.72)	1.59 (1.02–2.48)	2.67 (1.27–5.64)	0.003*
Sex	Male	1.00	1.65 (1.13–2.40)	1.57 (1.07–2.31)	2.71 (1.43–5.17)	0.003*
	Female	1.00	0.85 (0.57–1.25)	1.34 (0.91–1.97)	1.68 (0.87–3.25)	0.048*
BMI	<24 kg/m^2^	1.00	1.32 (0.90–1.94)	1.45 (0.98–2.14)	1.86 (0.86–3.99)	0.103
	≥24 kg/m^2^	1.00	0.97 (0.67–1.40)	1.44 (1.01–2.06)	2.27 (1.28–4.04)	0.001*
Diabetes	Yes	1.00	0.78 (0.36–1.70)	1.35 (0.61–2.97)	3.20 (0.99–10.3)	0.024*
	No	1.00	1.17 (0.88–1.55)	1.43 (1.08–1.90)	1.81 (1.09–3.01)	0.012*
Hypertension	Yes	1.00	1.07 (0.68–1.70)	1.39 (0.89–2.15)	1.81 (0.89–3.70)	0.065
	No	1.00	1.12 (0.81–1.55)	1.37 (0.98–1.92)	2.23 (1.22–4.08)	0.005**

^*^P < 0.05, ^**^P < 0.001, BMI: body mass index, LGA: low-grade albuminuria, UACR: urine albumin-to-creatinine ratio, Q1: quartile 1, Q2: quartile 2, Q3: quartile 3, Q4: quartile 4.
